# Asprosin as a Potential Link Between Vascular Inflammation and Disease Activity in Behçet's Disease

**DOI:** 10.1002/jcla.70216

**Published:** 2026-04-01

**Authors:** Zeynep Kaya, Mesude Seda Aydogdu, Cihat Ucar, Mehmet Nur Kaya, Sezgin Zontul, Elif Inanc, Servet Yolbas

**Affiliations:** ^1^ Division of Rheumatology, Department of Internal Medicine Istanbul Training and Research Hospital Istanbul Turkey; ^2^ Division of Rheumatology, Department of Internal Medicine Faculty of Medicine, Malatya Turgut Ozal University Malatya Turkey; ^3^ Department of Physiology Faculty of Medicine, Malatya Turgut Ozal University Malatya Turkey; ^4^ Division of Rheumatology, Department of Internal Medicine Faculty of Medicine, Balikesir University Balikesir Turkey; ^5^ Division of Rheumatology, Department of Physical Medicine and Rehabilitation Faculty of Medicine, Inonu University Malatya Turkey; ^6^ Division of Rheumatology, Department of Internal Medicine Faculty of Medicine, Inonu University Malatya Turkey

**Keywords:** asprosin, Behçet's disease, disease activity, uveitis, vascular inflammation

## Abstract

**Objective:**

Behçet's disease (BD) is a chronic multisystem vasculitis characterized by endothelial dysfunction and vascular inflammation. Asprosin is a recently identified adipokine implicated in endothelial injury and proinflammatory signaling. This study aimed to investigate serum asprosin concentrations in patients with BD and their relationship with disease activity and major clinical manifestations.

**Methods:**

This cross‐sectional case–control study enrolled 49 patients who fulfilled the International Criteria for Behçet's Disease and 48 healthy controls. Disease activity was stratified as mild, moderate, or severe based on organ involvement using Yosipovitch's Behçet's Disease Classification. Serum asprosin levels were quantified using an enzyme‐linked immunosorbent assay. Associations among asprosin levels, disease activity, clinical manifestations, and inflammatory markers were analyzed.

**Results:**

Serum asprosin concentrations were markedly elevated in individuals with Behçet's disease relative to healthy controls [12.18 (8.27–16.76) vs. 6.12 (4.76–8.61) ng/mL, *p* < 0.001] and increased incrementally as disease activity intensified (*p* < 0.001). Patients with vascular involvement and uveitis demonstrated markedly elevated asprosin levels compared to patients without these manifestations (*p* < 0.01). Correlation analysis revealed a strong positive association between serum asprosin levels and disease activity (*r* = 0.821, *p* < 0.001), and a moderate correlation with C‐reactive protein (CRP) levels (*r* = 0.431, *p* = 0.002). In multivariate analysis, both disease activity and CRP levels were independently associated with serum asprosin concentrations.

**Conclusion:**

Serum asprosin levels are elevated in BD and closely associated with disease activity, systemic inflammation, and major organ involvement, particularly vascular manifestations and uveitis. Asprosin may represent a potential biomarker of the inflammatory and vascular burden in BD.

## Graphical abstract

Serum asprosin levels are significantly elevated in patients with Behçet's disease compared to healthy controls. Asprosin concentrations increase progressively with disease activity and are strongly associated with vascular involvement and uveitis, suggesting a potential role as a biomarker of vascular inflammation and disease severity.

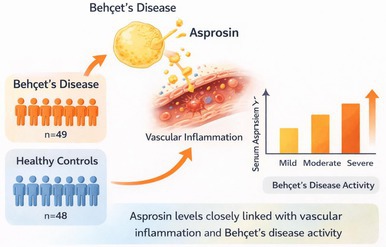

## 1 Introduction

Behçet's disease (BD) is a chronic, recurrent, multisystem inflammatory vasculitis characterized by repeated oral and genital ulcerations, ocular involvement, and various systemic manifestations involving both arteries and veins [1]. Endothelial dysfunction, chronic vascular inflammation, and thrombotic tendency are fundamental mechanisms in the pathogenesis of BD, often leading to vascular complications such as thrombophlebitis, arterial aneurysms, and occlusive vascular disease [2, 3].

Asprosin is a newly identified adipokine produced by adipose tissue during fasting and was initially described as a regulator of hepatic glucose release [4]. Beyond its metabolic effects, growing evidence suggests that asprosin is strongly associated with vascular pathologies. Experimental and clinical studies have demonstrated that asprosin contributes to endothelial dysfunction, induces phenotypic switching in vascular smooth muscle cells, and facilitates vascular remodeling by activating pro‐inflammatory signaling pathways such as TLR4–NF‐κB–NLRP3 [5]. Furthermore, asprosin has been implicated in endothelial‐to‐mesenchymal transition through TGF‐β signaling, thereby exacerbating peripheral arterial disease and vascular stiffness [6].

In this context, adipokines such as asprosin may represent a mechanistic link between metabolic pathways and immune‐mediated vascular inflammation, warranting investigation in systemic vasculitis as potential biomarkers of disease activity and vascular risk. Given that BD is a prototypical systemic vasculitis in which endothelial damage and vascular inflammation are central pathogenic mechanisms, the identification of novel biomarkers reflecting vascular involvement is of major clinical importance. Although endothelial dysfunction and surrogate markers of vascular injury (e.g., impaired flow‐mediated dilation, increased intima‐media thickness, oxidative stress markers) have been extensively studied in BD [7], the role of asprosin in this disease remains unexplored.

Therefore, this study aimed to compare serum asprosin levels between patients diagnosed with BD and age‐ and sex‐matched control subjects, evaluate the association between serum asprosin levels and disease activity, examine differences in asprosin levels according to major clinical manifestations, and identify determinants independently related to serum asprosin concentrations among patients with BD. Establishing such an association could provide insight into the pathogenic mechanisms of BD and potentially introduce asprosin as a novel biomarker for vascular risk stratification and disease monitoring.

## 2 Materials and Methods

In this cross‐sectional case–control study, participants aged between 18 and 65 years who satisfied the International Criteria for Behçet's Disease (ICBD) were included together with healthy control subjects matched for age and sex [8]. All participants were consecutively recruited from patients who presented to the outpatient clinic of the Malatya Training and Research Hospital between April 2025 and October 2025.

Inclusion criteria for the BD group were: age between 18 and 65 years, fulfillment of the ICBD, and provision of written informed consent. Inclusion criteria for the control group were age‐ and sex‐matched individuals without any history of inflammatory, autoimmune, or systemic vascular disease.

Exclusion criteria for both groups included pregnancy, active infection, known malignancy, diabetes mellitus, chronic kidney or liver disease, thyroid or other endocrine disorders, obesity (body mass index ≥ 30 kg/m^2^), and any acute inflammatory condition at the time of blood sampling. Healthy control subjects were additionally required to have no current or previous diagnosis of inflammatory or autoimmune disease.

The selected inclusion and exclusion criteria were designed to minimize potential confounding factors that could influence serum asprosin levels. Conditions known to affect metabolic status, systemic inflammation, or endothelial function, such as diabetes mellitus, obesity, endocrine disorders, and acute infections, were excluded to ensure a more homogeneous study population and to better isolate the relationship between BD activity and serum asprosin concentrations.

Disease status was assessed using Yosipovitch's Behçet's Disease Classification, an organ‐based disease severity classification system. In the present study, this classification was used to stratify patients according to disease activity based on the extent of organ involvement. Mild disease was defined as isolated mucocutaneous manifestations, moderate disease as the presence of ocular and/or articular involvement, and severe disease as major organ involvement including vascular or neurological manifestations. Although the Behçet's Disease Current Activity Form is widely used, Yosipovitch's classification was preferred in this study because it provides a practical, organ‐based framework that allows indirect stratification of disease activity according to disease severity in routine clinical practice.

Clinical manifestations, including vascular involvement, uveitis, mucocutaneous involvement, and articular involvement, were recorded as present or absent based on clinical and/or radiological confirmation. Demographic characteristics such as age, sex, body mass index (BMI), smoking status, and alcohol consumption were recorded for all the participants. In patients with BD, routine laboratory indices such as C‐reactive protein (CRP), erythrocyte sedimentation rate (ESR), and complete blood count were also documented.

Venous blood samples were collected from BD patients and healthy controls during routine venipuncture. Blood samples were collected in the morning after an overnight fast, and patients were clinically stable at the time of sampling. Serum samples were analyzed in a blinded manner, without knowledge of clinical status or disease activity, to minimize measurement bias. An additional 5 mL of venous blood was drawn to determine serum asprosin levels. The samples were centrifuged at 3000 rpm for 5 min and approximately 3 mL of serum was aliquoted into Eppendorf tubes. Serum specimens were stored at −20°C until further analysis.

Serum asprosin levels were measured using a commercially available enzyme‐linked immunosorbent assay (ELISA) kit (Cat. No: E4095HU; BT Lab, Shanghai, China), according to the manufacturer's protocol. All assays were conducted in duplicate and the average values were used for statistical analyses. The intra‐ and interassay coefficients of variation reported by the manufacturers were < 10%.

The study protocol was approved by the Ethics Committee of Malatya Turgut Ozal University Hospital (approval date: March 21, 2025; approval number: 2025/85). Written informed consent was obtained from all participants prior to enrollment. This study was conducted in accordance with the ethical principles of the Declaration of Helsinki.

## 3 Statistical Analysis

Statistical analyses were conducted using IBM SPSS Statistics for Windows, version 26.0 (IBM Corp., Armonk, NY, USA). The normality of continuous variables was evaluated using the Shapiro–Wilk test in combination with visual assessment of histogram distributions. Continuous data are reported as mean ± standard deviation (SD) when normally distributed and as median with interquartile range (IQR) when non‐normally distributed. Categorical variables are presented as frequencies and percentages.

Comparisons between patients with BD and healthy controls were performed using the Student's *t*‐test or Mann–Whitney *U* test according to data distribution, while categorical variables were analyzed using the chi‐square or Fisher's exact test, as appropriate. Within the BD group, comparisons of serum asprosin levels across disease activity categories were performed using the Kruskal–Wallis test. In cases where a statistically significant overall difference was observed, pairwise post hoc analyses were conducted using the Mann–Whitney *U* test with Holm's correction to determine intergroup differences. Comparisons of serum asprosin levels according to clinical features (vascular involvement, uveitis, mucocutaneous involvement, and articular involvement) were analyzed using the Mann–Whitney *U* test.

Correlation analyses were conducted to assess the relationship between serum asprosin levels and clinical or laboratory variables, including disease activity category, CRP level, ESR, age, and BMI exclusively in patients with BD using Spearman's rank correlation coefficient, given the non‐normal distribution of asprosin values. Multivariable linear regression analysis was performed to identify independent factors associated with serum asprosin concentration in patients with BD. Serum asprosin levels were log‐transformed before inclusion in the linear regression model to meet normality assumptions. Variables exhibiting a *p* value below 0.10 in univariable analyses were entered into the multivariable model. The results were presented as standardized beta coefficients (β) with 95% confidence intervals (CI). Model fit was assessed using the adjusted R^2^, and multicollinearity was evaluated by calculating the variance inflation factor (VIF). All statistical tests were two‐tailed, and a *p* value < 0.05 was considered statistically significant.

## 4 Results

This study included 49 patients with BD and 48 healthy controls. The demographic and clinical characteristics of the study population are summarized in Table 1. No statistically significant differences were observed between the patient and control groups with respect to age, sex distribution, BMI, smoking status, alcohol consumption, CRP level, or ESR (all *p* > 0.05). Serum asprosin concentrations were significantly elevated in patients with BD compared with those in healthy controls [12.18 (8.27–16.76) vs. 6.12 (4.76–8.61) ng/mL, *p* < 0.001].

**TABLE 1:** Demographic, clinical, and laboratory features of individuals with Behçet's disease and healthy controls.

Variables	Behcet's disease (*n* = 49)	Healthy controls (*n* = 48)	*p*
Age (years)	43.14 ± 12.22	43.77 ± 11.76	0.797
Female, *n* (%)	27 (55.1)	26 (54.2)	0.911
BMI (kg/m^2^)	25.81 ± 3.55	27.25 ± 3.77	0.056
Smoking, *n* (%)	17 (34.7)	22 (45.8)	0.286
Alcohol use, *n* (%)	3 (6.1)	4 (8.3)	0.678
CRP (mg/L)	3.00 (2.00–8.00)	4.50 (2.75–7.00)	0.537
ESR (mm/h)	9.00 (5.00–12.00)	9.00 (4.75–12.25)	0.991
Asprosin (ng/mL)	12.18 (8.27–16.76)	6.12 (4.76–8.61)	< 0.001

Abbreviations: CRP: C‐reactive protein, ESR: erythrocyte sedimentation rate, BMI: body mass index.

**FIGURE 1:**
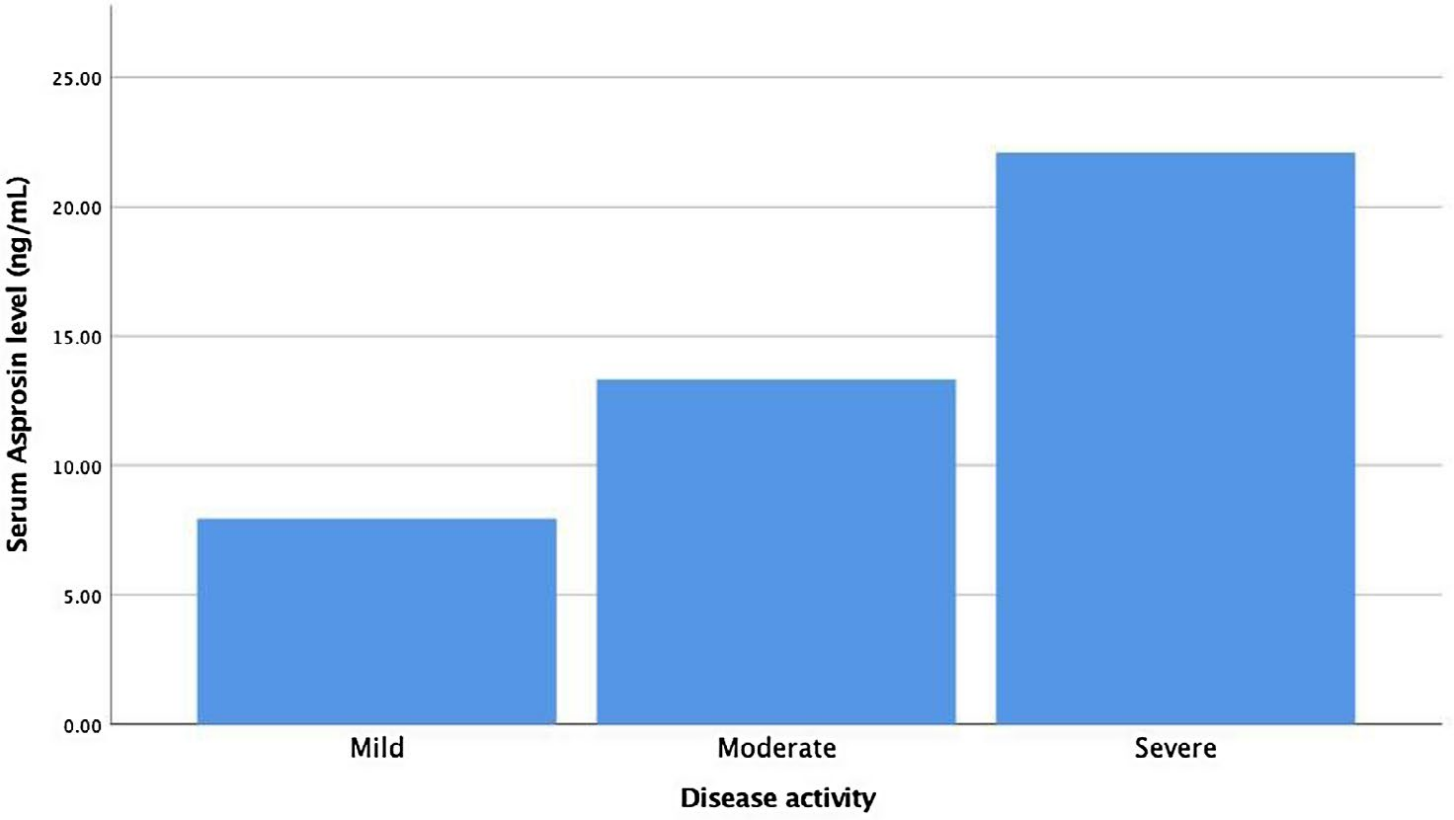
Distribution of serum asprosin levels according to disease activity categories in patients with Behçet's disease.

Based on disease activity, patients with BD were stratified into three groups: mild (*n* = 21), moderate (*n* = 11), and severe (*n* = 17) (Table 2). The serum asprosin levels progressively increased with increasing disease activity. Patients with mild disease activity had the lowest serum asprosin levels [7.78 (6.14–8.63) ng/mL], whereas those with moderate [13.57 (10.16–16.04) ng/mL] and severe disease activity [17.62 (15.31–25.99) ng/mL] had significantly higher concentrations (overall *p* < 0.001). Post hoc analyses revealed statistically significant differences among patients with mild disease compared to those with moderate‐to‐severe activity and between the moderate and severe groups. Serum asprosin levels increased progressively with disease activity severity (Figure 1).

**TABLE 2:** Serum asprosin levels according to disease activity.

A. Disease activity			
Activity	Asprosin (ng/mL), Median (IQR)	*p*	Post hoc
Mild (*n* = 21)	7.78 (6.14–8.63)		
Moderate (*n* = 11)	13.57 (10.16–16.04)		
Severe (*n* = 17)	17.62 (15.31–25.99)	< 0.001	Mild vs. Moderate–Severe; Moderate vs. Severe

Abbreviation: IQR: Interquartile range.

The serum asprosin levels according to major clinical features are presented in Table 3. Patients with vascular involvement had markedly higher serum asprosin levels than those without vascular involvement [29.09 (15.00–33.94) vs. 9.78 (7.46–12.73) ng/mL, *p* < 0.001]. Similarly, patients with uveitis exhibited significantly elevated asprosin concentrations compared to those without uveitis [16.07 (12.75–19.50) vs. 9.35 (7.12–12.88) ng/mL, *p* = 0.005]. In contrast, serum asprosin levels were not significantly associated with the presence of mucocutaneous or articular involvement (*p* = 0.450 and *p* = 0.580, respectively).

**TABLE 3:** Serum asprosin levels according to clinical features in patients with Behçet's disease.

Variables	Feature (+)	Feature (−)	*p*
Vascular involvement	29.09 (15.00–33.94)	9.78 (7.46–12.73)	< 0.001
Uveitis	16.07 (12.75–19.50)	9.35 (7.12–12.88)	0.005
Mucocutaneous involvement	11.82 (8.51–15.44)	10.94 (7.88–14.02)	0.450
Articular involvement	11.90 (8.39–14.22)	10.87 (7.92–13.95)	0.580

Correlation analyses identified a robust positive correlation between serum asprosin concentrations and disease activity category (*r* = 0.821, *p* < 0.001) (Table 4). Serum asprosin concentration also showed a moderate correlation with CRP level (*r* = 0.431, *p* = 0.002). No statistically significant associations were identified between serum asprosin levels and ESR, age, or BMI (all *p* > 0.05).

**TABLE 4:** Correlation of serum asprosin concentrations with clinical and laboratory parameters in patients with Behçet's disease.

Variables	Spearman's rho (r)	*p*
Disease activity category	0.821	< 0.001
CRP (mg/L)	0.431	0.002
ESR (mm/h)	0.170	0.242
Age (years)	−0.158	0.279
BMI (kg/m^2^)	−0.154	0.291

Abbreviations: BMI: body mass index, ESR: erythrocyte sedimentation rate, CRP: C‐reactive protein.

A multivariate linear regression model was used to determine the independent predictors of serum asprosin concentration in patients with BD (Table 5). Disease activity category (β = 0.68, 95% CI: 3.21–6.84, *p* < 0.001) and CRP levels (β = 0.24, 95% CI: 0.18–0.62, *p* = 0.011) were significantly related to higher serum asprosin levels. Age and body mass index were not independently associated with serum asprosin levels.

**TABLE 5:** Multivariate linear regression analysis of determinants of serum asprosin concentrations in Behçet's disease patients.

Independent variable	*β* (Standardized)	95% CI	*p*
Disease activity category	0.68	3.21–6.84	< 0.001
CRP (mg/L)	0.24	0.18–0.62	0.011
Age (years)	−0.07	−0.09–0.03	0.321
BMI (kg/m^2^)	−0.06	−0.28–0.11	0.404

Abbreviations: CI: confidence interval, BMI: body mass index, CRP: C‐reactive protein.

## 5 Discussion

In the present study, serum asprosin concentrations were significantly elevated in patients with BD compared to healthy controls and were strongly associated with disease activity, systemic inflammation, and major organ involvement, particularly vascular manifestations and uveitis. These findings add to the expanding literature indicating the involvement of asprosin in inflammatory and immune‐mediated conditions and provide novel insights into its potential involvement in the pathophysiology of BD.

The experimental data provide important mechanistic insights into the biological effects of asprosin. Orton et al. showed that asprosin activates multiple intracellular pathways, including mTOR, NOTCH, and WNT signaling, which are critically involved in angiogenesis, cellular metabolism, and immune regulation [9]. These pathways are also central to the pathogenesis of chronic inflammatory and autoimmune diseases, which supports the mechanistic basis of the findings observed in our cohort.

A substantial body of clinical and experimental evidence has linked elevated asprosin levels with cardiovascular inflammation and vascular dysfunction. Increased serum asprosin concentrations have been reported in patients with hypertension, coronary artery disease, and acute coronary syndrome and are significantly correlated with cardiovascular risk and disease severity [10, 11, 12, 13, 14]. Experimental studies have further demonstrated that asprosin promotes vascular remodeling and inflammation via superoxide signaling and TLR4–NFκB–mediated NLRP3 inflammasome activation [15, 16, 17]. These findings suggest that asprosin may act as a systemic pro‐inflammatory mediator, amplifying inflammatory cascades beyond metabolic tissues, which is consistent with our results on inflammatory rheumatic diseases.

Vascular involvement is one of the most severe and prognostically important manifestations of BD and is driven by endothelial dysfunction, neutrophil hyperactivation, and exaggerated inflammatory responses. Oxidative stress is another key mechanism that links asprosin to inflammatory disease activity. Elevated asprosin levels have been associated with increased oxidative stress markers in systemic lupus erythematosus and acute myocardial infarction [18, 19]. Conversely, experimental interventions targeting asprosin have been shown to attenuate oxidative stress and neointima formation following vascular injury [20], highlighting its potential role as a modifiable inflammatory mediator. These observations align with our findings demonstrating a close relationship between asprosin levels and inflammatory burden. Although CRP is a nonspecific inflammatory marker, the absence of a detectable difference between the two groups may be related to the cross‐sectional design of the study and the absence of acute disease flares at the time of sampling.

Recent studies have examined the involvement of asprosin in rheumatological and inflammatory disorders. Sipahioglu et al. reported increased serum asprosin levels during Familial Mediterranean Fever attacks, suggesting that asprosin levels may reflect acute inflammatory activity [21]. In contrast, Uslu et al. observed lower serum asprosin levels in patients with fibromyalgia syndrome [12], a condition characterized primarily by central sensitization rather than systemic inflammation. This discrepancy suggests that asprosin elevation may be specifically associated with active inflammatory processes rather than chronic pain syndromes without overt inflammation, which supports the interpretation of our findings.

Uveitis is another major cause of morbidity in patients with BD and reflects intense intraocular inflammation. To date, no clinical or experimental study has directly investigated the relationship between asprosin and uveitis. Nevertheless, indirect evidence suggests a potential link, as pro‐inflammatory cytokines induced by asprosin, particularly interleukin‐6 (IL‐6) and tumor necrosis factor‐α (TNF‐α), play pivotal roles in the pathogenesis of immune‐mediated ocular inflammation [22, 23]. The significantly higher serum asprosin levels observed in patients with uveitis in our cohort constitute a novel finding and suggest that asprosin may be involved in inflammatory pathways that contribute to ocular involvement in BD. This observation further distinguishes our study from previous asprosin research and highlights a previously unexplored area.

In contrast, mucocutaneous and articular involvement were not associated with significant differences in serum asprosin levels. This finding suggests that asprosin is more closely linked to systemic and organ‐threatening inflammation than to localized or relatively mild disease manifestations. These findings support the hypothesis that asprosin is particularly relevant for severe disease phenotypes.

From a clinical perspective, the association between serum asprosin levels and disease activity suggests that asprosin may serve as a complementary biomarker to traditional inflammatory markers. If confirmed in longitudinal studies, serum asprosin measurement could potentially aid in disease monitoring and risk stratification, particularly in patients with vascular involvement.

The present study had several limitations that warrant consideration. First, the modest sample size and single‐center setting may have restricted the external validity of the results. Second, the cross‐sectional design did not allow for inferences regarding causality or temporal associations between serum asprosin levels and disease activity. Longitudinal studies assessing asprosin levels during disease flares and remission periods are needed to clarify their dynamic behavior. Third, although disease activity was stratified using Yosipovitch's organ‐based Behçet disease severity classification, the use of additional validated disease activity indices might have strengthened the assessment of disease activity. Fourth, vascular involvement was analyzed as a composite variable, and subgroup analyses according to vascular subtype were not performed because of the limited sample size. Finally, we did not evaluate other cytokines or endothelial dysfunction markers that could help elucidate the mechanistic pathways linking asprosin to vascular and ocular inflammation.

## 6 Conclusion

In conclusion, patients with BD exhibit increased serum asprosin levels that are closely linked to disease activity and markers of systemic inflammation. Although previous experimental and clinical studies have demonstrated a relationship between asprosin levels and vascular inflammation in various inflammatory and cardiometabolic conditions, this study is one of the first clinical investigations of serum asprosin concentration in BD and demonstrated its association with vascular involvement. Furthermore, there is currently no published data evaluating the relationship between asprosin and uveitis in any clinical setting. The increased asprosin concentrations detected in patients with BD‐associated uveitis constitute a novel and potentially significant addition to the literature. These results suggest that asprosin may play a key role in the inflammatory pathways underlying major organ involvement in BD and highlight the need for further mechanistic and longitudinal studies to clarify its pathogenic significance and potential utility as a biomarker of disease activity.

## Author Contributions

All authors contributed substantially to the conception and design of the study: acquisition, analysis, and interpretation of data and drafting or critically revising the manuscript for important intellectual content. All authors read and approved the final version of the manuscript for submission.

## Funding

This research did not receive any specific grant from funding agencies in the public, commercial, or not‐for‐profit sectors.

## Ethics Statement

The study protocol was approved by the Ethics Committee of Malatya Turgut Ozal University Hospital (approval date: March 21, 2025; approval number: 2025/85). All procedures performed in this study were in accordance with the ethical standards of the Institutional Research Committee and principles of the Declaration of Helsinki. Written informed consent was obtained from all the participants prior to their inclusion in the study.

## Consent

The authors have nothing to report.

## Conflicts of Interest

The authors declare no conflicts of interest.

## Data Availability

The data that support the findings of this study are available on request from the corresponding author. The data are not publicly available due to privacy or ethical restrictions.
